# Current and Future Replacement and Opportunity Costs of Family Caregiving for Older Americans With and Without Dementia

**DOI:** 10.1093/geroni/igaf049

**Published:** 2025-05-31

**Authors:** Stipica Mudrazija, María P Aranda

**Affiliations:** Department of Health Systems and Population Health, School of Public Health, University of Washington, Seattle, Washington, USA; USC Edward R. Roybal Institute on Aging, Suzanne Dworak-Peck School of Social Work, University of Southern California, Los Angeles, California, USA

**Keywords:** Forgone earnings, Lost productivity, Projections, Replacement cost, Unpaid income tax

## Abstract

**Background and Objectives:**

Family caregivers in the United States provide substantial value of unpaid care to older adults while less recognized are the employment-related costs they endure and the trajectory of these costs. We estimate the replacement cost of unpaid family caregiving to U.S. adults aged 70 and older with and without dementia and the opportunity costs of forgone earnings and lost productivity between 2011 and 2060.

**Research Design and Methods:**

We match caregivers to older adults from the National Study of Caregiving with similar noncaregivers from the Panel Study of Income Dynamics. We use population projections alongside current and historical data on educational attainment, wages, inflation, and average wages for in-home care aides to approximate total replacement and opportunity costs.

**Results:**

Current annual replacement cost of unpaid family care is between $96 and $182 billion, 44% of which is accounted for by dementia caregiving. By 2060, it will increase to $277–571 billion, and 53% will be for dementia caregiving. The opportunity costs of forgone earnings and productivity loss, however, will grow faster, increasing from current levels of $107 billion and $26 billion to $380 billion and $102 billion, respectively, in 2060. Projections show that opportunity costs of family caregiving will be increasingly borne by caregivers of older adults with dementia and racial/ethnic minoritized caregivers.

**Discussion and Implications:**

As the employment-related opportunity costs of family caregiving for older adults are on a trajectory to become increasingly similar in value to associated replacement costs of unpaid care, policymakers, health insurance payers, and employers should focus on supporting unpaid family caregivers to remain attached to the labor force through efforts such as strengthening paid family leave options, expanding consumer-directed in-home services options, and offering increased work flexibility.

**Translational Significance:**

An increasing number of Americans are expected to provide care to older family members, which may adversely affect their ability to work. We estimate the replacement costs of unpaid family caregiving to U.S. adults aged 70+ with and without dementia, and associated employment-related opportunity costs through 2060, finding that these opportunity costs will grow faster. Policies should focus on the dual role of family caregivers as paid workers in the labor market and unpaid caregivers at home. Addressing the caregiving needs of all families is a key issue facing all Americans, especially populations that are vulnerable to economic impacts.

## Background and Objectives

Caregiving to older family members with physical and/or cognitive disabilities entails high levels of hands-on care and complex medical tasks, negotiating external long-term services and supports (LTSS), balancing employment-related responsibilities, and bearing out-of-pocket costs (1). More than 17 million adult Americans, of whom almost two-thirds are women, provide care to someone (overwhelmingly, family members) 65 years of age or older, which is expected to grow significantly in the future (2). Much has been said regarding the importance of valuing unpaid caregiving in the United States, especially given that two-thirds of older adults rely solely on unpaid family care, and many more receive at least some of it (3). The value of unpaid care is generally derived from the cost it would take to replace it with paid care, that is, the replacement cost, which is the approach that we also take in this paper. However, there has been less attention given to economic costs related to various burdens associated with providing unpaid care (4,5). Chief among these are opportunity costs, which include forgone earnings for unpaid caregivers, lost productivity for their employers, or lost federal income tax revenue, among others. These costs are not uniformly distributed across caregivers, and evidence suggests that disparities persist with regard to economic burdens facing different population groups (6,7).

In this context, our paper has 3 main objectives: (a) to estimate current and future replacement costs of unpaid family caregiving to U.S. adults aged 70 and older and opportunity costs of forgone earnings and lost productivity between 2011 and 2060, (b) to compare these costs between 2 groups of caregivers, namely those caring for a loved one living with dementia and without dementia, and (c) to compare costs by educational attainment and race and ethnicity. A key motivation for this paper is to estimate future costs of caregiving, identify groups which may be at greatest risk for severe financial consequences, and to inform evidence-based workplace and government policies and programs. Importantly, the term “caregiver” is not consistently defined in the extant literature as some reports are more or less inclusive based on consanguine ties (or not), hours of caregiving, types of care tasks, or subjective reports of caregivers or care recipients, among others. We rely on an authoritative national survey that defines caregivers to older adults (henceforth, family caregivers) based on older respondents’ reports. Another important consideration is that although we make a distinction between paid work (ie, employment, including self-employment) and unpaid caregiving, both entail “work,” one that is paid through employment-based means and the other one that refers to caregiving work without pay.

### Replacement Costs of Unpaid Family Caregiving

Literature has long focused on valuing unpaid caregiving and estimating the cost to replace it. One estimate puts the total lifetime unpaid care value per adult aged 50 and older at $168 000 (4). However, estimates of the total annual replacement cost of unpaid care differ substantially depending on the assumed number of caregivers, which varies based on the type of unpaid support that is recognized as caregiving, and the choice of the occupational wage, most commonly for professional caregivers, to value it. Upper bound estimates suggest unpaid caregiving may be worth as much as half a trillion dollars or more (8,9). With the projected increase of the over-65 U.S. adult population from 52 million in 2018 to 95 million by 2060 (10), we expect significant increases in the need for caregiving.

### Opportunity Costs of Unpaid Family Caregiving

Opportunity costs of caregiving stem from employment-related impacts such as having to stop working or reducing work hours, changing jobs or positions (often with lower pay), and foregoing promotions or merit increases (11,12). Forgone earnings for caregivers aged 20–64 were $70 billion in 2013 and will increase to $150 billion (in 2013 constant dollars) by midcentury (11).

Not all opportunity costs are borne by caregivers. Other costs include, among others, productivity loss (cost to employers because of employees’ decreased work productivity while physically present at work or absent from work), lost income tax payments (that would have been collected by the government), or increased government outlays for caregivers (due to their lower earnings and/or worse health). The focus, however, has traditionally remained on forgone earnings, presumably due to limited data availability. Thus, analytic advancements, especially as they relate to drawing from multiple data sources to estimate a broader set of costs associated with future valuations of unpaid care provision, bring us closer to more realistic assessments of costs and benefits of caregiving.

Opportunity costs vary mostly by educational attainment and race/ethnicity. Higher costs are typically borne by people with college degrees, who generally earn substantially more than those without college degrees, and this difference continues to increase. College education has grown uninterruptedly over the past half century, a trend that has further accelerated in recent years (7). Moreover, research shows divergent trends in real wages for workers with different educational attainment (13). Racial/ethnic minoritized caregivers on average provide substantially more hours of care than non-Hispanic White caregivers, which may be partly related to their constrained ability to pay for care. For example, non-Hispanic Black caregivers bear higher costs of caregiving than non-Hispanic White caregivers because they are more likely to provide over 40 h of care per week, care for someone living with dementia (14), and provide higher levels of assistance with activities of daily living (15). Hispanic caregivers face similar challenges with greater care demands, less assistance from external service providers (15), and limited use of formal care services such as nursing homes and hospice (16) than non-Hispanic White caregivers. Non-Asian racial/ethnic minoritized family caregivers are also more likely to report being “highly financially strained” (17). Black caregivers’ out-of-pocket spending on care-related costs is more than 34% of their annual income, compared to 14% for non-Hispanic White caregivers (17). Hispanic women report spending 56% of household income on caregiving compared to the average caregiver at 38% and are more likely than any other group to report that caregiving affects work (18).

The outlined differences suggest that estimates of opportunity cost are strongly affected by assumptions researchers make about the factors included and the method of their valuation.

### Valuation of Caregiving Costs and Dementia

Although the economic cost of caregiving affects all caregivers, not all caregiving is alike. Differences in its valuation emerge particularly when care recipients have dementia and are from racial/ethnic minoritized populations (6,7,19). Ample evidence supports that caregiving for persons living with dementia entails significant challenges related to management of memory and behavioral symptoms, comprehension and communication processes, interactions with healthcare (eg, LTSS) providers, prevention of injury, neglect and abuse, and facilitation of autonomy in decision making, legal and financial transactions, among others (1,20,21). Additionally, compared to nondementia caregivers, those caring for family members with dementia are more likely to report providing more hours of care, work in highly intensive or physically demanding care contexts, and report higher rates of distress and caregiving strain (12). Thus, the economic costs associated with caregiving for persons living with dementia are substantially higher than for caregivers of family members without dementia. Currently, total costs associated with treating Alzheimer’s disease are estimated at $321 billion annually (22), and family caregivers bear 70% of the lifetime cost of providing care to loved ones with dementia (15).

Given the outlined multidimensional nature of economic costs associated with unpaid caregiving and differences related to providing care to persons with and without dementia, in this paper, we estimate the current and future replacement and opportunity costs of unpaid family care to older Americans with and without dementia by race and ethnicity from 2011 to 2060. We focus on the major opportunity costs, including forgone earnings (before tax and excluding earnings-related benefits) and lower work productivity. To calculate work productivity, we combine reports of absenteeism (ie, productivity lost due to missing time at work) and presenteeism (ie, not performing work while being physically present at it), following the methodological approach outlined in the NHATS/NSOC documentation (23). In supplementary analysis, we also analyze lost federal income tax, which does not increase the overall opportunity costs, but has redistributional implications at a societal level.

The use of multidimensional economic cost measures is key to ensuring a more robust consideration of the economic implications of unpaid caregiving and is the first step toward building a more comprehensive societal cost–benefit model of family caregiving. This information should guide future program and policy development efforts to mitigate adverse financial impacts of caregiving without disincentivizing care provision to family members who overwhelmingly prefer to be cared for by family in their own residence (24).

## Research Design and Methods

### Data

Data on family caregivers come from the 2011, 2015, 2017, and 2021 waves of the National Study of Caregiving (NSOC), a nationally representative supplement to the National Health and Aging Trends Study (NHATS), which collects information on U.S. family caregivers to Medicare beneficiaries aged 65 and older. Medicare covers all adults aged 65 and older (as well as people with disabilities), except those who did not meet the program’s work requirement and are not married to someone who met the work requirement, and foreign-born people who are neither U.S. citizens nor permanent residents. NSOC includes data on the type, duration, and intensity of help caregivers provided, possible effects of caregiving on caregivers, support services they may have used, and employment-related information. Caregivers are identified by the NHATS respondents during the interview and subsequently contacted. If a respondent identifies more than 5 caregivers, the survey randomly selects 5 caregivers to interview. Sample weights account for survey nonresponse as well as differential probabilities of sample selection.

Our analytic sample pools data on all adult family caregivers (including spouses/partners, own children, stepchildren, children-in-law, siblings, siblings-in-law, parents, stepparents, parents-in-law, grandchildren, aunts, uncles, nephews, nieces, cousins, stepchildren’s children, children-in-law’s children, and other relatives) to NHATS respondents aged 70 and older from the 4 available waves on NSOC. We exclude those younger than 70 because 2 more recent NHATS waves of our interest do not include them, and we want to have a balanced pooled sample with respect to the age representation over time. Since the prevalence of dementia and other major chronic conditions is comparatively low at these ages (25); however, this is not a major drawback. We distinguish between caregivers for older adults classified as likely having dementia and for caregivers of those without dementia based on an algorithm utilizing 3 sources of information: self or proxy report of dementia diagnosis; results of the AD8 Dementia Screening Interview administered to proxy respondents; and tests of memory, orientation, and executive function, administered to NHATS respondents (26). The initial analytic sample for this study includes 6 124 caregivers, including 1 775 from the first survey wave (813 with dementia), 1 759 from the second wave (682 with dementia), 1 446 from the third wave (465 with dementia), and 1 144 from the fourth wave (318 with dementia). While the NSOC was not originally designed as a longitudinal survey, it elicits information on caregivers of NHATS sample persons who are followed longitudinally. Therefore, there is a limited number of caregivers who provided information more than once. In such cases, we use information from the first wave when a caregiver participated in the survey and the older adult was at least 70 years old. Also, in 2017, NSOC started with a longitudinal component, which collected interviews from caregivers originally identified in 2015, but in this analysis, we focus strictly on the cross-sectional samples of NSOC across the 4 waves of interest. The NSOC data is supplemented with relevant information on NHATS participants, including their age, race/ethnicity, and dementia status.

Data on noncaregivers come from the 2013 wave of the Panel Study of Income Dynamics (PSID), a nationally representative longitudinal panel survey of U.S. families. This sample includes 8 615 heads and spouses who reported no provision of help to parents. The key information on the provision of time support to parents comes from the 2013 Rosters and Transfers module and is supplemented with sociodemographic and health information from the main survey, including information on labor force status and hours worked. While the design of the PSID questionnaire allows identifying nonhelpers, it is not appropriate for identifying helpers to parents (or other family members) for health or functioning reasons as it does not distinguish this type of help from other types of practical help. Therefore, it is not possible to analyze employment-related impacts of caregiving using the PSID data only.

Because the PSID questionnaire elicits information on the provision of time support to parents only, it is possible that some persons in the noncaregiver sample provide care to others. However, as about 3-quarters of working-age caregivers to older adults are children, and the prevalence of caregiving for this population is below 7%, no more than 2% of the noncaregiver sample might be affected. Given the observed negative impact of caregiving on the likelihood of employment and hours worked, this could result in a marginal downward bias of the estimates.

The reference periods for the NSOC and PSID are somewhat different. While the NSOC asks about paid work in the prior week, PSID asks about paid work at the time of the interview. Similarly, the NSOC asks about hours worked in the prior week, whereas PSID asks about average hours worked in the prior year. Given that the NSOC does not make any explicit reference to overtime hours, the analysis in this study relies on a more conservative PSID measure of hours worked that leaves out any overtime hours worked.

We use data from multiple sources to produce estimates of the economic costs of providing care through 2060. Projections of demographic trends by age and race and ethnicity come from the 2017 National Population Projections. Historic trends in educational attainment by race and ethnicity come from the Current Population Survey (CPS) (27). We also use CPS for historic information on average earnings. Consumer Price Index retroactive series (R-CPI-U-RS) information from the Bureau of Labor Statistics is used to adjust price levels over time to real (2021) U.S. dollars. Information on federal income tax payments by adjusted gross income levels for tax year 2020 (filed in 2021), utilized in supplementary analysis (Supplementary Table 7), comes from the Internal Revenue Service (28). Data on home health and personal care aides’ mean hourly wage in 2022 come from the Bureau of Labor Statistics (29).

### Analytic Approach

To estimate current replacement costs of unpaid care, lost wages and productivity, and lost federal income tax payments, we perform multivariate-distance matching of caregivers to older adults from NSOC with their noncaregiver peers from PSID, stratifying the matching by race and ethnicity (non-Hispanic White and others) and by educational attainment (college degree vs less than college degree). The samples are matched on sociodemographic and health characteristics comparable across the 2 surveys: age (in years); gender; marital status (married/partnered vs unmarried); homeownership; self-rated health (excellent/very good, good, and fair/poor); and having any living siblings. We request an exact match with respect to caregivers’ gender and marital status given the strong evidence of systemic differences in caregiving by those characteristics. Gender differences in caregiving feature particularly prominently in the literature as it relates to the likelihood, type, and intensity of care provided to older family members (30). These results are used alongside information from other data sources to derive current and future estimates of economic costs of providing care to persons with dementia.

With regards to matching NSOC caregivers to older adults with their noncaregiver peers from the PSID, the matching procedure and results are outlined in Supplementary Material (Supplementary Tables 1 and 2).

Stratification of estimates by educational attainment and race and ethnicity is important because, based on historic trends and existing projections, the U.S. population (including people in need of care and unpaid caregivers) will change substantially along these 2 dimensions. College education has grown uninterruptedly over the past half century, a trend that has accelerated in recent years (7). Given the acceleration in college degree attainment over time and inherent uncertainty, we test several alternative scenarios for the expected change in educational attainment through 2060. The results we present are from our medium estimate, which relies on trends since 2000. Results from the low estimate (average gain in college degree attainment since 1976) and high estimate (average gain since 2010) are available on request.

Accounting for educational attainment change over time is key given that the opportunity cost of caregiving is much higher for those with at least a college degree who are more likely to be employed and earn higher hourly wages than those without a degree. Moreover, research shows divergent trends in real wages for workers with different educational attainment (13). Changes in racial and ethnic composition of the population are also important inasmuch as the intensity of unpaid care provision varies significantly by race and ethnicity as previously discussed.

We account for differential trends in real wage growth by educational attainment. Using data from CPS, we calculate that over the 2 decades preceding the onset of COVID-19 (between 1999 and 2019), real wages increased by a total of 4.3% for those with less than a college degree, and 8.3% for those with at least a college degree. We extrapolate these real wage growth rates into the future.

Various input numbers for caregiving projections ranging from productivity loss for employed caregivers and employment-related characteristics of the matched PSID sample of noncaregivers to projected college degree attainment by race and ethnicity, real wage growth factor by college attainment, and projected population of family caregivers by dementia status of care recipients, educational attainment, and race and ethnicity are available in Supplementary Material (Supplementary Tables 3–6).

Finally, we make several simplifying assumptions in our projections. First, we assume that the overall provision of care will increase proportionately with the increase in the number of people living with dementia and their need for LTSS. This implies that the unmet care needs will also increase at the same pace. Second, we assume unchanged preference for informal caregiving, disregarding the possibility that the relative prices of alternatives to informal caregiving—primarily home care and nursing homes—may change and shift the demand for informal caregiving independently of population characteristics. If such a change would occur, the estimate could be either too high (if the relative price of alternatives declines) or too low (if it increases). Furthermore, we assume that the relative prices of family and paid care will remain broadly constant and that the profiles of people needing care for each subgroup of caregivers will remain constant over time.

## Results

Between 2011 and 2021, about 17.6 million family caregivers provided care to adults aged 70 and older, of which 5.9 million, or one-third, were caring for someone with dementia (Table 1). Somewhat over 27% of family caregivers were from racial/ethnic minoritized groups, including 33% of caregivers to persons with dementia. About 7.5 million worked in addition to providing care. The number of family caregivers will increase to more than 47 million by 2060, and during the same period, the number of employed caregivers will increase even faster, reaching over 21 million. Racial/ethnic minoritized caregivers will be substantially overrepresented among families caring for a loved one with dementia (56% vs 43% for nondementia caregivers in 2060).

**Table 1.** Current and Projected Population of Family Caregivers, by Dementia Status of Care Recipients and Race and Ethnicity

	Dementia		No Dementia		Total
	Non-Hispanic Whites	Racial/ethnic minoritized groups	Non-Hispanic Whites	Racial/ethnic minoritized groups	
**All family caregivers**					
2011–2021	3.9	2.0	8.9	2.9	17.6
2030	6.1	4.1	14.2	5.8	30.1
2040	7.8	6.3	16.5	8.2	38.7
2050	8.4	8.3	16.7	10.0	43.4
2060	8.1	10.4	16.5	12.3	47.4
**Workers family caregivers**					
2011–2021	1.7	0.9	3.5	1.3	7.5
2030	2.6	2.0	5.6	2.7	12.9
2040	3.4	3.0	6.6	3.9	16.9
2050	3.8	4.0	6.8	4.8	19.3
2060	3.7	5.0	6.8	5.9	21.4

*Notes*: National Study of Caregiving, 2011–2021; National Health and Aging Trends Study, 2011–2021; 2017 National Population Projections; Current Population Survey, 1976–2022; authors’ estimates.

Because there is no universally accepted method of approximating the replacement costs of care, in Table 2, we present 2 estimates: the lower-bound estimate based on the federal minimum hourly wage, and the upper-bound estimate, based on the average hourly cost of paid professional caregivers (ie, home health and personal care aides). Results suggest that, in the 2011–2021 period, the replacement cost of unpaid family care to adults aged 70 and older was somewhere between $96 billion and $182 billion. By 2060, the annual replacement cost of unpaid care will increase to somewhere between $277 billion and $571 billion. Over the same period, the share of caregiving provided to older adults with dementia will increase from 45% to 53%, while the share of racial/ethnic minoritized caregivers will rise from 38% to 62%. Racial/ethnic minoritized caregivers are disproportionately caring for older adults with dementia, and this difference is projected to increase in the future.

**Table 2.** Current and Future Value of Family Caregiving Using Federal Minimum Wage and Replacement Wage for Professional Caregivers, by Dementia Status of Care Recipient and Race and Ethnicity ($2021bn)

	2011–2021	2030	2040	2050	2060
**Minimum wage**					
*Dementia*					
Non-Hispanic Whites	24.0	35.8	45.0	47.9	45.3
Racial/ethnic minoritized groups	19.2	40.7	61.8	81.9	102.0
*No dementia*					
Non-Hispanic Whites	35.5	55.3	62.8	62.1	60.2
Racial/ethnic minoritized groups	17.3	34.1	47.6	57.3	69.2
Total—minimum wage	96	165.9	217.2	249.2	276.7
**Replacement wage**					
*Dementia*					
Non-Hispanic Whites	45.5	69.4	89.0	96.7	93.4
Racial/ethnic minoritized groups	36.5	78.8	122.3	165.3	210.4
*No dementia*					
Non-Hispanic Whites	67.4	107.1	124.2	125.4	124.1
Racial/ethnic minoritized groups	32.9	66.1	94.1	115.7	142.8
Total—replacement wage	182.3	321.4	429.6	503.1	570.7

*Notes*: National Study of Caregiving, 2011–2021; National Health and Aging Trends Study, 2011–2021; 2017 National Population Projections; Current Population Survey, 1976–2022; Bureau of Labor Statistics, 2023, 2023b; authors’ estimates.

However, many family caregivers are unable to continue paid work or have to work fewer hours than they otherwise would. Table 3 shows that in the 2011–2021 period, family caregivers lost about $107 billion annually in earnings. Eighty percent was attributable to lower labor force participation, while 20% was due to fewer hours worked among caregivers who were employed. Somewhat less than one-third of the $107 billion in lost earnings was accounted for by caregiving to older adults with dementia. Our estimates of the cost of forgone earnings attributable to racial/ethnic minoritized persons are higher than those found in the previous work using the NSOC data (10). The difference is mostly related to the composition of the caregiver sample, which was limited to adults aged 20–64 in the previous study, whereas in this study, we include older caregivers, too. This is important because many caregivers, especially those in their mid-to-late 60s, may retire earlier than planned or decrease their hours of paid work, which is a major source of forgone earnings. Moreover, that study uses 2013 dollars while we use 2021 dollars, which creates an appearance of a larger difference than it is. Adjusting for this difference, for example, our total annual cost of forgone earnings would be less than $92 billion in 2013 dollars (as opposed to $107 billion in 2021 dollars).

**Table 3.** Current and Future Value of Forgone Earnings for Family Caregivers, by Dementia Status of Care Recipient, and Race and Ethnicity ($2021bn)

	2011–2021	2030	2040	2050	2060
**Dementia**					
*Non-Hispanic Whites*					
Lower labor force participation rate	18.3	31.0	42.8	50.1	52.2
More part-time versus full-time employment	6.5	10.7	14.9	17.5	18.3
*Racial/ethnic minoritized groups*					
Lower labor force participation rate	7.6	17.4	28.5	40.8	55.1
More part-time versus full-time employment	1.9	4.3	7.1	10.2	13.8
Total—dementia	34.3	63.4	93.3	118.6	139.4
**No dementia**					
*Non-Hispanic Whites*					
Lower labor force participation rate	47.9	84.1	105.9	116.0	124.6
More part-time versus full-time employment	11.1	18.3	22.3	23.7	24.8
*Racial/ethnic minoritized groups*					
Lower labor force participation rate	11.3	24.8	39.7	54.7	75.6
More part-time versus full-time employment	2.2	5.0	8.1	11.3	15.7
Total—no dementia	72.5	132.2	176	205.7	240.7
***Total***	***106.8***	***195.6***	***269.3***	***324.3***	***380.1***

*Notes*: National Study of Caregiving, 2011–2021; National Health and Aging Trends Study, 2011–2021; Panel Study of Income Dynamics, 2013; 2017 National Population Projections; Current Population Survey, 1976–2022; Bureau of Labor Statistics, 2023; authors’ estimates.

By 2060, the cost of forgone earnings is expected to reach $380 billion, with the share of forgone earnings accounted for by care provision to older adults with dementia increasing to 37%. Over the next 4 decades, the overall share of forgone earnings attributable to racial and ethnic minorities will increase from 22% to 42%, and from 28% to 49% among caregivers to older adults with dementia.

While these numbers reflect the increasing number of older adults’ caregivers and their changing composition, especially as it relates to their educational profile and the related labor market prospects, it is also important to understand how these changes will affect the financial well-being of individual caregivers. As shown in Table 4, the amount of per-person forgone earnings for racial/ethnic minoritized dementia caregivers is somewhat lower than for non-Hispanic Whites. The difference amounted to somewhat over $1 400 per year in the 2011–2021 period ($6 323 for non-Hispanic Whites and $4 875 for racial/ethnic minoritized groups) and will grow to almost $2 000 ($8 647 vs $6 652) per year in 2060. Among nondementia caregivers, the difference by race and ethnicity decreases over time, from under $2 000 ($6 661 vs 4 692) in recent years, to just over $1 600 in 2060 ($9 027 vs $7 398).

**Table 4.** Per Person Amount and Percentage of Current and Future Forgone Earnings for Family Caregivers, by Dementia Status of Care Recipient and Race and Ethnicity

	2011–2021	2030	2040	2050	2060
**Amount (in 2021 dollars)**					
*Dementia*					
Non-Hispanic Whites	6 323	6 893	7 433	8 017	8 647
Racial/ethnic minoritized groups	4 875	5 266	5 685	6 145	6 652
*No dementia*					
Non-Hispanic Whites	6 661	7 220	7 780	8 382	9 027
Racial/ethnic minoritized groups	4 692	5 176	5 852	6 591	7 398
**% earnings**					
*Dementia*					
Non-Hispanic Whites	11.3	11.3	11.4	11.5	11.6
Racial/ethnic minoritized groups	12.5	12.7	13.0	13.2	13.4
*No dementia*					
Non-Hispanic Whites	12.1	12.2	12.3	12.3	12.4
Racial/ethnic minoritized groups	12.0	12.6	13.3	14.0	14.7

*Notes*: National Study of Caregiving, 2011–2021; National Health and Aging Trends Study, 2011–2021; Panel Study of Income Dynamics, 2013; 2017 National Population Projections; Current Population Survey, 1976–2022; Bureau of Labor Statistics, 2023; authors’ estimates.

Moreover, as a percentage of earnings, racial/ethnic minoritized caregivers lose as much (for nondementia caregivers) or more (for dementia caregivers) than non-Hispanic White caregivers. Compared with the 2011–2021 period, non-Hispanic White caregivers of persons with and without dementia lost on average 11.3% and 12.1% of their earnings, respectively, yet by 2060 these percentages will increase only moderately by 0.3 percentage points. The increases over the same period for racial/ethnic minoritized populations will be more pronounced, growing by 0.9 percentage points for dementia caregivers (12.5% vs 13.4%) and by 2.7 percentage points for nondementia caregivers (12% vs 14.7%).

Forgone earnings, however, are not the only employment-related cost. Absenteeism (missing work) and presenteeism (decreased efficiency at performing work tasks) attributable to family caregiving represent a cost for employers. And, although lower work productivity may not have an immediate negative impact on caregivers’ earnings, it may adversely affect them in the long run by hurting their prospects of raises and promotions. Moreover, a part of forgone earnings would have been collected by the federal government through income taxes. While this does not represent an additional financial loss and therefore does not increase the overall opportunity costs, it has societal resource allocation implications inasmuch as federal taxes are used to pay for public programs, including health care and community supports for people with medical, psychiatric, and neurocognitive comorbidities.

In the 2011–2021 period, the annual value of lost productivity for employed caregivers was, on average, almost $26 billion, and it is expected to almost quadruple by 2060 (Table 5). Over the same period, the share of lost productivity attributable to dementia caregivers will increase from 48% to 56%, and the share borne by racial/ethnic minoritized caregivers will increase from 24% to 42%.

**Table 5.** Current and Future Value of Productivity Loss for Employed Family Caregivers, by Dementia Status of Care Recipient and Race and Ethnicity ($2021bn)

	2011–2021	2030	2040	2050	2060
*Dementia*					
Non-Hispanic Whites	8.8	15.4	22.1	26.9	28.9
Racial/ethnic minoritized groups	3.7	8.4	14.1	20.5	28.1
*No dementia*					
Non-Hispanic Whites	10.9	19.2	24.5	27.3	29.7
Racial/ethnic minoritized groups	2.5	5.4	8.4	11.2	15.2

*Notes*: National Study of Caregiving, 2011–2021; National Health and Aging Trends Study, 2011–2021; Panel Study of Income Dynamics, 2013; 2017 National Population Projections; Current Population Survey, 1976–2022; Bureau of Labor Statistics, 2023; Income Revenue Service, 2022; authors’ estimates.

## Discussion and Implications

We set out to estimate current and future replacement cost of unpaid family care to adults 70 years old and older with and without dementia, and to calculate related opportunity costs of forgone earnings, lower productivity, and lost federal income tax payments for family caregivers, their employers, and the government, respectively. We find that the replacement cost of unpaid family caregiving is valued at $96–182 billion annually, which will roughly triple by 2060. Over half of this future care will be provided to older adults with dementia, and almost two-thirds by racial/ethnic minoritized caregivers.

The cost of forgone earnings is also large, and combined with the lower productivity of employed caregivers, is roughly comparable to the replacement cost of unpaid care. Caregivers to persons with dementia account for roughly one half of the care provided (and related replacement costs) and lost productivity, but only about one third of forgone earnings. This is attributable to the compositional differences between dementia and nondementia family caregivers; in particular, dementia caregivers are older and more likely to be retired or out of the labor force.

As the U.S. population continues to age, unpaid family caregiving will increase sharply. This increase will outpace the growth of the older adult population because of the growing share of racial/ethnic minoritized older adults who rely on family care more than non-Hispanic White older adults. Simultaneously, employment-related opportunity costs of unpaid family care will grow even faster than its replacement costs due to the expected increase in the share of college-educated caregivers whose lost paid work hours and productivity are particularly costly. Both the replacement costs of unpaid family care and employment-related opportunity costs of caregiving will be increasingly attributable to dementia caregiving, as the population with dementia is growing fast and, on average, requires more intensive care.

What these results show is that caregiving comes at a great financial cost of forgone earnings for family caregivers. There are also additional losses for their employers (lower productivity). Lost earnings are increasingly borne by racial/ethnic minoritized persons and dementia caregivers as their numbers continue to increase faster than that of non-Hispanic Whites and nondementia caregivers, and as they provide more intensive care.

This implies that increasing attention should be given to the provision or subsidy of older-adult care by the public sector. Current programs are largely financed by Medicaid, which is based on strict income eligibility standards. This leaves most caregivers without robust support, including many near poor from racial/ethnic minoritized groups. Thus, increased financial support through cash payments, tax incentives, caregiver credits, and paid respite care, among others, would help buffer the economic stresses that the unpaid care of older adults can impose (5,31–33).

### Limitations

There are limitations related to our data and analytic assumptions. Dementia classification in NHATS is not based on clinical classification but on other information, including respondent (or proxy) report of dementia diagnosis and various tests that measure respondents’ memory, temporal orientation, judgment, and executive function. This raises the prospect of dementia misclassification, although comparisons of dementia prevalence in NHATS and Health and Retirement Study, including its Aging, Demographics, and Memory Study supplement, show substantial accordance in estimated prevalence, with some overestimation before age 80 and underestimation after age 80 (26).

Furthermore, when valuing unpaid family care, we rely on minimum and replacement wages. A more refined approach would be to use only replacement wages, but to account for possible efficiency gains (ie, fewer hours of care for the same level of care) from using professional care. However, we lack data allowing such calculation. A further cautionary note, though, is that even if we had such data, it would likely be inaccurate inasmuch as it would not account for other, intrinsic aspects of the value of family caregiving, such as its emotional value to care recipients.

Data limitations also prevent us from assessing the impact of caregiver burden on government expenditures for health and social programs, calculating the additional loss of fringe benefits related to caregivers’ forgone earnings, or caregivers’ forgone contributions to savings, retirement, and investment accounts. Moreover, it could be argued that not every hour of care has equal value or same implications on employment-related costs: some caregiving activities may be more burdensome than others, and hours of care that can be shifted at caregivers’ discretion presumably impact their ability to work much less than the hours provided on a fixed schedule and/or frequently.

Because of limited sample size and the need for reliable estimates, we are not able to distinguish between different racial and ethnic groups. Based on population trends and insights from other studies, it is clear that the main driver of predicted strong growth in unpaid family care and related costs is the rapid increase in the older Hispanic population (10). Older Hispanic adults are substantially more likely than non-Hispanic Whites to have dementia (34), and their family caregivers provide more hours of help and report more adverse impacts on their ability to remain attached to the labor force and productive at work. Similarly, we did not have a sufficient sample size to stratify our analysis further by gender, although women provide more intensive care and often terminate employment early due to caregiving (35). However, since their future share in the population is going to remain roughly constant, gender-specific estimates would likely have no distributional impact on our projections, while decreasing the robustness of the sample. However, this could change if, for example, substantial divergence occurs in future labor force participation trends of women and men, but there is no indication that this is likely to happen.

Because of our primary focus on caregivers and, relatedly, on caregiving that occurs and is reliably measured, we do not attempt to calculate the cost to meet all caregiving needs of older adults, which would also include the cost of unmet needs. However, we recognize that unmet needs are an important element of the overall demand for care, and any changes to its future trajectory would have implications for paid and unpaid care provision. Past reviews have also shown the importance of identifying unmet caregiver needs such as self-care activities, information and skill-building, and long-term services (36). For this reason, future research would benefit from studying historic trends in unmet needs of older adults and their caregivers and establishing more accurately how unmet needs might change in response to changes in demand for care, price of paid care, and supply and characteristics of potential unpaid caregivers.

We further recognize that, if the average annual duration of episodic care by unpaid family caregivers was longer than 1 month, we would be underestimating the total volume of episodic care provided. The existing literature provides little guidance in this regard as it is primarily focused on chronic conditions and largely silent as it relates to the characteristics of unpaid home episodic care such as its prevalence, duration, type, and intensity (37). Based on industry standards for episodic care management (ECM) that recommend having patients discharged from hospitals or skilled nursing facilities in ECM for up to 30 days following discharge, it is possible to infer that typical episodic care lasts no longer than 1 month. However, we cannot exclude the possibility that it may last somewhat longer.

Focusing on future estimates, we identify several limitations. Among the most important ones is the role of changing gender norms around caregiving. While these norms have changed over time and they may change in the future, we have no evidentiary foundation to project if, when, and how exactly they might change, which precludes us from incorporating this information into our calculations.

Moreover, population trends imply that a growing share of older adults will be without any potential family caregiver available. Yet, declining geographic mobility in the United States (27) might offset this by increasing the share of older adults with family members living nearby, a precondition for care provision at a regular schedule and/or over longer periods of time. It is unclear what the net impact of these trends will be, but addressing care needs when no caregiver is available is paramount.

Beyond these limitations in calculating current and future replacement and opportunity costs, we recognize that there are likely long-run “secondary” costs of caregiving that cannot be assessed with the existing data. We already mentioned that lower productivity of employed caregivers could adversely affect their prospects for career advancement. Similarly, their need to stop working or scale back their hours worked can be detrimental to their employers inasmuch as they find themselves unable to replace caregivers with other workers of similar productivity. Indeed, even if they are successful in finding equally suitable replacements, there are costs involved with identifying, hiring, and onboarding such workers, making this a potentially costly transition. While we are unable to quantify these “secondary” impacts of caregiving for caregivers’ and their employers’ costs, we recognize them as important cost elements that the future work needs to account for as data become available.

Finally, it could be argued that caregivers’ loss of leisure is also an opportunity cost. While we do not have a reliable way of valuing leisure in this analysis, adding it to the opportunity cost of caregiving would certainly further alter the difference between replacement and opportunity cost. Similarly, given the inherently limited nature of time as a resource, to the extent that caregiving for older parents may take away time to spend taking care of their own children or grandchildren, or other activities such as volunteering, could further increase the opportunity costs of caregiving.

Despite these limitations, the present study provides important insights into the benefits and costs—both personal and societal—of relying on family as a primary source of care for older adults with dementia and other health conditions. Results suggest that this is associated with an increasingly high cost of forgone earnings, which negatively affects the well-being of caregivers and their families. Projections show that costs of family caregiving will be increasingly borne by caregivers of older adults with dementia and racial/ethnic minoritized caregivers.

Our results suggest that the scope is expanding for programs and policies to support dual roles of paid workers and caregivers for persons caring for their loved ones. Employers should be encouraged and incentivized to support caregivers with flexible work schedules, wellness days, robust employee-assistance programs, more progressive time-off options, and voluntary leave transfer programs. Health insurance innovations—whether in the private or public sector—should provide robust support for affordable, consumer-directed, in-home care services provided by trained staff to relieve complex caregiving demands, including respite time. Future research should examine the effects of the economic costs on caregivers from diverse backgrounds to address health equity concerns. Policy efforts should be aligned with the comprehensive actions recommended by the National Strategy to Support Family Caregivers over the life course for all U.S. Americans, including efforts to further expand and improve paid family leave options (38).

Finally, in a larger context, our research provides further evidence of the importance and value of unpaid family caregiving as an economic activity. While we recognize that the difficulties in tracking unpaid caregiving for older adults (as well as for children) or volunteer activities may have historically precluded recognizing this important segment of the economy, we believe that our work demonstrates that increased availability of nationally representative data in the United States that collects information on some of the key home-produced unpaid services is making it feasible to start thinking about the ways to include them in the calculations of the gross domestic product, even if primarily as a part of a supplementary measure (ie, satellite account) that allows for adjustment of the traditional measure (39). Such change would increase the visibility of these economic activities as well as the people who engage in them, who are disproportionately women, minoritized populations, and other traditionally underprivileged groups in society.

## Supplementary Material

igaf049_suppl_Supplementary_Tables_S1-S7

## Data Availability

This study uses public versions of the NHATS/NSOC and PSID data. NHATS/NSOC data are produced and distributed by www.nhats.org with funding from the National Institute on Aging and the Office of the Assistant Secretary of Planning and Evaluation, Department of Health and Human Services (grants U01AG032947, R01AG054004, and R01AG062477). PSID data are produced and distributed by the Survey Research Center, Institute for Social Research, University of Michigan, Ann Arbor, MI, partly supported by the National Institutes of Health (grants R01 HD069609 and R01 AG040213) and the National Science Foundation (awards SES 1157698 and 1623684). These data are available free of charge to any registered user who agrees to abide by the rules set out by the institutions collecting and storing them. The NHATS/NSOC and PSID do not allow third-party distribution of their data by anyone, but any researcher interested in replicating analyses can obtain them directly by registering for data access. All other information that we utilized, such as the U.S. Census Bureau data, is freely available for download at sources that we listed. The study was not preregistered.
