# Supplementary material for: Current and Future Replacement and Opportunity Costs of Family Caregiving for Older Americans With and Without Dementia
Source: Innov Aging. 2025 May 31;9(6):igaf049. doi: 10.1093/geroni/igaf049 (PMC12257478; doi:10.1093/geroni/igaf049)
Supplement: igaf049_suppl_Supplementary_Tables_S1-S7 [file igaf049_suppl_supplementary_tables_s1-s7.docx]

***Innovation in Aging* Supplementary Material: Mudrazija & Aranda. Current and Future Replacement and Opportunity Costs of Family Caregiving for Older Americans With and Without Dementia.**

**Matching Methodology and Example**

Information on caregivers to older adults with and without dementia comes from a 2011-2021 pooled sample of the National Study of Caregiving (NSOC). Given that our NSOC sample does not have information on non-caregivers, we needed to identify a suitable sample of non-caregivers from another nationally-representative data source that has information on caregiving and employment and does not limit the age of adult caregivers. The only data source satisfying all the criteria that we identified was PSID, and more precisely, its 2013 Rosters and Transfers module. However, because the NSOC sample consists of caregivers to NHATS respondents, that is, people Medicare-eligible adults aged 65 and older, which we further limited to 70 and older, as younger ages are not represented across all survey waves, and because there are other characteristics that vary nonrandomly between caregivers and non-caregivers such as their sex or marital status, we needed to match the NSOC and PSID samples to be able to compare employment-related outcomes.

Because we have a small number of covariates available across both NSOC and PSID that we consider important, and we want to make sure that our matches are as close as possible across all of them, we use Mahalanobis (multivariate-distance) matching. This matching methodology directly calculates the distance between treated (caregivers) and untreated (non-caregivers) individuals using the covariance matrix and directly accounting for correlations across all variables of interest. The NSOC and PSID samples are matched on the following sociodemographic and health characteristics: age (in years); gender; marital status (married/partnered vs. unmarried); homeownership; self-rated health (excellent/very good, good, and fair/poor); and having any living siblings. Additionally, we request an exact match with respect to caregivers’ sex and marital status, because of the overwhelming prior empirical evidence of differences in caregiving likelihood (and intensity) based on these characteristics.^29^

Importantly, we stratify the matching by race and ethnicity (non-Hispanic white and others) and by educational attainment (college degree vs. less than college degree), thereby producing four sets of estimates for each employment-related outcome of interest (e.g., likelihood of employment for college-educated non-Hispanic whites, likelihood of employment for college-educated Hispanics and other non-whites, etc.). Stratification of estimates by educational attainment and race and ethnicity is critical for our analysis because of the assumption, based on both the historical trends and the existing projections, that the US population, including those in need of care and their unpaid caregivers, will change most along these two dimensions. Over the coming decades, older Americans and their caregivers will be, on average, much better educated and much more likely to be from minoritized population groups, which is why we need to account explicitly for differences in the employment-related burdens across these four subgroups of Americans. This is also the reason why we do not stratify our sample further by sex since the sex composition of population (and, therefore, the female-to-male ratio) will likely change very little if at all in the coming decades, and is therefore sufficient to have sex as one of the critical matching characteristics rather than a stratification characteristic. This assumption could be violated only if there would be some unforeseen dramatic development that would result in selectively altering the sex ratio in the United States, such as the impact of World War II was on the decline of male population in some European countries (e.g., Russia or Soviet Union at the time) or the impact of one child policy at its peak on the excess number of male children relative to female children in China. Stratifying the analysis further by gender or any other characteristic that exhibits no potential for a clear sustained trend of change during the projection horizon, therefore, would come at a price of having to produce many more matching estimates based on ever smaller subsamples, resulting in increasingly unreliable estimates without obvious gains with respect to the need for such information for projecting future trends.

With this context in mind, we next aim to give an insight into the results of our matching procedure and the quality of information that we then utilize in calculating employment-related costs of caregiving. First, in Supplementary Table 1, we show the full set of estimates of differences between caregivers and matched non-caregivers in the likelihood of employment and the likelihood of full-time employment. The results suggest that caregiving is related to a noticeable decline in the likelihood of employment and full-time employment among caregivers relative to non-caregivers. For the likelihood of employment, having a college degree is associated with a larger difference in the likelihood of employment, especially for minoritized populations. For full-time employment, however, we find divergent trends by race and ethnicity, with larger differences in the likelihood for college-educated minoritized groups and non-college-educated non-Hispanic whites. There is no clear difference between dementia and non-dementia caregivers, however, in the impact of caregiving on these two employment-related outcomes.

**Supplementary Table 1. Estimated differences in the likelihood of employment and the likelihood of full-time employment among workers between caregivers and matched non-caregivers**

|  | **Dementia** | | | |
| --- | --- | --- | --- | --- |
|  | **No college degree** | | **College degree** | |
|  | Non-Hispanic whites | Racial/ethnic minoritized groups | Non-Hispanic whites | Racial/ethnic minoritized groups |
| Difference in the likelihood of employment | -0.079 | -0.090 | -0.088 | -0.123 |
| Difference in the likelihood of full-time employment among workers | -0.109 | -0.082 | -0.082 | -0.117 |
|  |  |  |  |  |
|  | **No dementia** | | | |
|  | **No college degree** | | **College degree** | |
|  | Non-Hispanic whites | Racial/ethnic minoritized groups | Non-Hispanic whites | Racial/ethnic minoritized groups |
| Difference in the likelihood of employment | -0.086 | -0.064 | -0.109 | -0.164 |
| Difference in the likelihood of full-time employment among workers | -0.112 | -0.045 | -0.052 | -0.123 |

Source: National Study of Caregiving, 2011-2021; National Health and Aging Trends Study, 2011-2021; Panel Study of Income Dynamics, 2013; authors’ estimates.

Each of the estimates in Supplementary Table 1 is a result of a separately fitted matching procedure. To illustrate the mechanics of it, in Supplementary Table 2 we further present results for one of these estimates, for the likelihood of employment for non-college-educated minoritized dementia caregivers.

**Supplementary Table 2. Mahalonobis-distance kernel matching results for the likelihood of employment, and means and variances of raw and matched data for non-college educated minoritized dementia caregivers and non-caregivers.**

| Matching results | | |  |  |  |
| --- | --- | --- | --- | --- | --- |
|  | Treated | | Controls | | Bandwidth |
|  | Yes | No | Yes | No |  |
| Matched sample size | 731 | 19 | 2648 | 238 | 1.033468 |
|  | Coefficient | Standard error | t value | *p*>t | 95% confidence interval |
| ATT | -0.090 | 0.028 | -3.240 | 0.001 | {-0.144 - -0.035] |

| Means and variances of raw and matched data | | | | | | | |  |  |
| --- | --- | --- | --- | --- | --- | --- | --- | --- | --- |
|  | Raw data | | | |  | Matched data | | | |
|  | Treated | | Controls | |  | Treated | | Controls | |
|  | Mean | Variance | Mean | Variance |  | Mean | Variance | Mean | Variance |
| Age (in years) | 57.3 | 203.9 | 45.7 | 260.3 |  | 57.3 | 197.5 | 55.8 | 189.1 |
| Female | 0.68 | 0.22 | 0.58 | 0.24 |  | 0.67 | 0.22 | 0.67 | 0.22 |
| Married/partnered | 0.47 | 0.25 | 0.51 | 0.25 |  | 0.47 | 0.25 | 0.47 | 0.25 |
| Homeownership | 0.49 | 0.25 | 0.40 | 0.24 |  | 0.48 | 0.25 | 0.48 | 0.25 |
| Self-rated health (ref. Excellent/Very good) |  |  |  |  |  |  |  |  |  |
| Good | 0.31 | 0.21 | 0.34 | 0.22 |  | 0.31 | 0.22 | 0.31 | 0.22 |
| Fair/Poor | 0.26 | 0.19 | 0.27 | 0.20 |  | 0.27 | 0.20 | 0.27 | 0.20 |
| Any living siblings | 0.56 | 0.25 | 0.90 | 0.09 |  | 0.58 | 0.24 | 0.58 | 0.24 |

Source: National Study of Caregiving, 2011-2021; National Health and Aging Trends Study, 2011-2021; Panel Study of Income Dynamics, 2013; authors’ estimates.

The results show that there are, on average, almost four PSID matched non-caregivers (i.e., controls) used for every NSOC caregiver (i.e., treated). Only about 2.5% of caregivers (19 out of 750) were not used because of the lack of appropriate matching controls. Following the matching procedure, we get a matched sample that has a much more similar profile than before the matching, with only minor remaining differences in the average age. The same procedure is repeated for each combination of race and ethnicity and education for dementia and non-dementia caregivers across the two outcomes of interest (likelihood of employment and full-time employment) for a total of 16 outcomes shown in Supplementary Table 1. These detailed results are available on request.

**Other Supplementary Material to Research Design and Methods Section**

**Supplementary Table 3. Estimated productivity loss as a share of employment time for employed caregivers**

|  | **Dementia** | | | |
| --- | --- | --- | --- | --- |
|  | **No college degree** | | **College degree** | |
|  | Non-Hispanic whites | Racial/ethnic minoritized groups | Non-Hispanic whites | Racial/ethnic minoritized groups |
| Difference in the likelihood of employment | -0.059 | -0.083 | -0.109 | -0.132 |
|  |  |  |  |  |
|  | **No dementia** | | | |
|  | **No college degree** | | **College degree** | |
|  | Non-Hispanic whites | Racial/ethnic minoritized groups | Non-Hispanic whites | Racial/ethnic minoritized groups |
| Difference in the likelihood of employment | -0.046 | -0.042 | -0.059 | -0.049 |

Source: National Study of Caregiving, 2011-2021.

**Supplementary Table 4.** **Employment-related characteristics of the matched sample of non-caregivers**

|  | **Dementia** | | | | **No dementia** | | | |
| --- | --- | --- | --- | --- | --- | --- | --- | --- |
|  | **No college degree** | | **College degree** | | **No college degree** | | **College degree** | |
|  | Non-Hispanic whites | Racial/ethnic minoritized groups | Non-Hispanic whites | Racial/ethnic minoritized groups | Non-Hispanic whites | Racial/ethnic minoritized groups | Non-Hispanic whites | Racial/ethnic minoritized groups |
| **Average Hours** | 37.1 | 37.0 | 36.8 | 39.9 | 36.4 | 36.7 | 36.1 | 40.9 |
| Full-Time Hours | 43.8 | 42.5 | 45.5 | 42.8 | 43.7 | 42.4 | 45.5 | 43.3 |
| Part-Time Hours | 19.7 | 20.4 | 18.2 | 22.9 | 19.1 | 20.4 | 17.4 | 23.4 |
| **Weeks Employed** | 48.4 | 48.3 | 48.8 | 49.5 | 47.8 | 47.6 | 48.5 | 49.4 |
| **Hourly Wage** | 24.3 | 19.2 | 44.0 | 30.1 | 24.6 | 18.6 | 43.1 | 30.8 |

Source: National Study of Caregiving, 2011-2021; National Health and Aging Trends Study, 2011-2021; Panel Study of Income Dynamics, 2013; authors’ estimates.

**Supplementary Table 5.** **Projected college degree attainment by race and ethnicity and real wage growth factor by college attainment, 2030-2060**

|  | **2030** | **2040** | **2050** | **2060** |
| --- | --- | --- | --- | --- |
| **College degree** |  |  |  |  |
| Non-Hispanic whites | 42.2 | 48.1 | 53.9 | 59.7 |
| Racial/ethnic minoritized groups | 29.9 | 35.0 | 40.0 | 45.1 |
| **Real wage growth factor** |  |  |  |  |
| No college degree | 1.021 | 1.043 | 1.065 | 1.087 |
| College degree | 1.041 | 1.083 | 1.127 | 1.172 |

Source: Current Population Survey, 1976-2022; authors’ estimates.

**Supplementary Table 6.** **Current and projected population of family caregivers, by dementia status of care recipients, educational attainment and race and ethnicity**

|  | **Dementia** | | | | **No dementia** | | | |  |
| --- | --- | --- | --- | --- | --- | --- | --- | --- | --- |
|  | **No college degree** | | **College degree** | | **No college degree** | | **College degree** | |  |
|  | Non-Hispanic whites | Racial/ethnic minoritized groups | Non-Hispanic whites | Racial/ethnic minoritized groups | Non-Hispanic whites | Racial/ethnic minoritized groups | Non-Hispanic whites | Racial/ethnic minoritized groups | **Total** |
| ***All family caregivers*** |  |  |  |  |  |  |  |  |  |
| 2011-2021 | 2.6 | 1.6 | 1.4 | 0.4 | 5.5 | 2.2 | 3.3 | 0.6 | 17.6 |
| 2030 | 3.5 | 3.1 | 2.6 | 1.0 | 7.8 | 4.3 | 6.4 | 1.5 | 30.1 |
| 2040 | 4.0 | 4.5 | 3.8 | 1.8 | 8.1 | 5.7 | 8.4 | 2.5 | 38.7 |
| 2050 | 3.8 | 5.6 | 4.6 | 2.7 | 7.2 | 6.5 | 9.4 | 3.5 | 43.4 |
| 2060 | 3.2 | 6.5 | 4.9 | 3.9 | 6.2 | 7.4 | 10.3 | 4.9 | 47.4 |
| ***Workers family caregivers*** |  |  |  |  |  |  |  |  |  |
| 2011-2021 | 1.0 | 0.7 | 0.7 | 0.2 | 1.9 | 1.0 | 1.6 | 0.4 | 7.5 |
| 2030 | 1.3 | 1.4 | 1.3 | 0.5 | 2.7 | 1.8 | 3.0 | 0.9 | 12.9 |
| 2040 | 1.5 | 2.1 | 1.9 | 0.9 | 2.8 | 2.5 | 3.8 | 1.4 | 16.9 |
| 2050 | 1.4 | 2.5 | 2.4 | 1.4 | 2.5 | 2.8 | 4.3 | 1.9 | 19.3 |
| 2060 | 1.2 | 2.9 | 2.5 | 2.1 | 2.1 | 3.2 | 4.7 | 2.7 | 21.4 |

Source: National Study of Caregiving, 2011-2021; National Health and Aging Trends Study, 2011-2021; 2017 National Population Projections; Current Population Survey, 1976-2022; authors’ estimates.

**Opportunity Cost of Lost Federal Income Tax Revenue**

**Supplementary Table 7. Current and future value of federal tax revenue loss for employed family caregivers, by dementia status of care recipient and race and ethnicity ($2021bn)**

|  | **2011-2021** | **2030** | **2040** | **2050** | **2060** |
| --- | --- | --- | --- | --- | --- |
| *Dementia* |  |  |  |  |  |
| Non-Hispanic whites | 1.7 | 2.9 | 4.1 | 4.9 | 5.1 |
| Racial/ethnic minoritized groups | 0.6 | 1.3 | 2.2 | 3.2 | 4.4 |
| *No dementia* |  |  |  |  |  |
| Non-Hispanic whites | 4.0 | 7.2 | 9.1 | 10.1 | 10.9 |
| Racial/ethnic minoritized groups | 0.9 | 1.9 | 3.2 | 4.4 | 6.2 |

Source: National Study of Caregiving, 2011-2021; National Health and Aging Trends Study, 2011-2021; Panel Study of Income Dynamics, 2013; 2017 National Population Projections; Current Population Survey, 1976-2022; Bureau of Labor Statistics, 2023; Income Revenue Service, 2022; authors’ estimates.
